# Endocrine Disruption as a Mediator of Declining Semen Quality in Smokers

**DOI:** 10.3390/cells14171345

**Published:** 2025-08-29

**Authors:** Birupakshya Paul Choudhury, Sandipan Das, Kushal Kumar Kar, Petr Slama, Adriana Kolesarova, Israel Maldonado Rosas, Shubhadeep Roychoudhury

**Affiliations:** 1 Department of Life Science and Bioinformatics, Assam University, Silchar 788011, India; birupakshyapc@gmail.com (B.P.C.); sandipankls@gmail.com (S.D.); 2 Department of Zoology, Cachar College, Silchar 788001, India; 3 Mediland Hospital and Research Centre, Silchar 788002, India; kushalkar@hotmail.com; 4 Laboratory of Animal Immunology and Biotechnology, Department of Animal Morphology, Physiology and Genetics, Faculty of AgriSciences, Mendel University in Brno, 61300 Brno, Czech Republic; petr.slama@mendelu.cz; 5 AgroBioTech Research Centre, Slovak University of Agriculture in Nitra, 949 76 Nitra, Slovakia; adriana.kolesarova@uniag.sk; 6 Institute of Applied Biology, Faculty of Biotechnology and Food Sciences, Slovak University of Agriculture in Nitra, 949 76 Nitra, Slovakia; 7 Citmer Reproductive Medicine, Mexico City 11520, Mexico; imaldonado@citmer.mx

**Keywords:** male infertility, reproduction, cigarette smoking, sperm quality, reproductive hormones, hormonal regulation, multiple linear regression

## Abstract

Background: Cigarette smoking is one of the most common lifestyle and environmental risk factors for male infertility. Although smoking has been implicated in male fertility decline, the association between endocrine disruption and semen quality reduction remains underexplored in smokers. This study demonstrates the impact of reproductive hormones on the modulation of semen quality in infertile men. Methods: Eighty infertile men participated in this observational study. They were interviewed for environment and lifestyle factors, following which their semen and four reproductive hormones, viz, follicle-stimulating hormone (FSH), luteinizing hormone (LH), prolactin (PRL), and testosterone, were analyzed. A comparative analysis between non-smokers and smokers highlighted notable differences in hormone levels and semen quality. To explore the relationships between reproductive hormones and semen quality parameters, correlation and multiple linear regression analyses were conducted. Results: Smokers exhibited a lower percentage of live sperm (*p* = 0.0000) and a reduction in normal morphology. Furthermore, elevated levels of FSH, LH, and PRL were found among smokers (*p* = 0.0000). Notably, heightened levels of LH and PRL were linked to a decreased percentage of live sperm cells, while increased LH alone significantly impacted sperm concentration. FSH showed a negative correlation with both live sperm cells (r = −0.50) and total sperm count (r = −0.46). In contrast, testosterone levels demonstrated a positive association with normal sperm morphology (r = 0.47). Conclusion: Cigarette smoking disrupts the regulation of reproductive hormones, which further impacts semen quality. This study provides insights into the potential impact of smoking on semen quality through hormonal mechanisms.

## 1. Introduction

Infertility is one of the primary health concerns around the world, with prevalence ranging between 12.6% and 17.5% among couples of reproductive age [1]. Global data indicate a steady rise in male infertility across the last few decades, marked by a drastic reduction in sperm concentration and total sperm count [2]. However, apart from the long-standing issues that result in a reduction of male reproductive health, other relevant threats include a global decline in semen parameters, less sexual intercourse (planned), and family planning among younger adults, fewer or contradictory investigations in male reproductive health, and inadequate knowledge about infertility [3]. Rapid changes in environmental conditions and the growing prevalence of unhealthy lifestyles have contributed to a steady rise in reproductive disorders and infertility [4]. It has been observed that lifestyle issues, such as smoking, alcohol intake, and obesity, are responsible for the steady decline of male fertility. Direct and even passive smoking is associated with a reduction in semen parameters such as semen volume, sperm concentration, vitality, motility, and morphology [5].

Smoking is a significant modifiable lifestyle factor associated with the decline of male fertility, with evidence linking it with a dose-dependent decline of sperm concentration [6]. The global smoking prevalence stands at 17%, with 21% of smokers residing in high-income countries, 17% in middle-income countries, and 10% in low-income countries [7]. Cigarette smoke contains toxic metabolites such as nicotine and heavy metals, which are absorbed into the systemic circulation and accumulate in the seminal plasma, leading to oxidative stress (OS) and sperm DNA damage [8]. A recent meta-analysis provided ample evidence for the association of smoking with reduced sperm count, motility, and morphology [9]. Toxic compounds in tobacco smoke can cross the blood–testis barrier and induce OS-mediated damage to spermatozoa [10].

Alternatively, exposure to environmental toxins such as endocrine-disrupting chemicals (EDCs) aggravates the ill effects of other lifestyle stressors, such as cigarette smoke, on the reproductive system. EDCs are exogenous compounds often found in pesticides, pharmaceuticals, and synthetic food products, which interfere with endocrine homeostasis, mimic, and block hormone signaling, leading to disruptions in physiological processes [11,12]. EDCs act via nuclear receptors, non-nuclear steroid hormone receptors, and nonsteroid receptors, such as serotonin/dopamine receptors, among others. Potential sources of EDCs include industrial byproducts such as polychlorinated biphenyls (PCBs), polybrominated biphenyls (PBBs), bisphenol A (BPA), phthalates, personal care products, pesticides such as dichlorodiphenyltrichloroethane (DDT), pharmaceutical agents, etc. [11,13]. Existing evidence suggests that exposure to EDCs results in the direct as well as transgenerational decline in semen quality. EDCs can interfere with the binding of hormones to the steroid receptors and act as antiandrogenic agents [11,13,14]. Moreover, studies have reported higher levels of reproductive hormones and poorer semen parameters in men who were exposed to EDCs, such as BPA and phthalates. Oxidative stress-induced impairments resulting from EDCs have been implicated in perturbations of both hormonal regulation and sperm cell function [15]. Emerging concerns suggest that the co-exposure to smoking and widespread EDCs may act synergistically to aggravate reproductive anomalies, though this interaction remains largely unexplored.

There are reports indicating that smoking interferes with the secretory function of Sertoli and Leydig cells, which alters spermatogenesis and normal sperm health [16]. In a recent cross-sectional study, it was reported that smoking significantly reduced semen volume and total sperm count [17]. Apart from rendering cellular damage, smoking further interferes with the pituitary–gonadal axis, leading to endocrine disruption [18], although there are contradictory outcomes too [19].

Smoking further increases the secretion of prolactin (PRL) from the anterior pituitary and blocks the release of dopamine [20]. While some studies report elevated levels of total testosterone and luteinizing hormone (LH) in smokers [21], others find no significant differences in LH, follicle-stimulating hormone (FSH), or testosterone among smoking men [8]. It has been reported that nicotine from cigarette smoke results in a rise in LH and PRL levels [22]. However, another study reported increased levels of total testosterone and LH in smokers [23]. A separate study observed elevated levels of LH and FSH and reduced testosterone in smokers [24]. Thus, the interplay of reproductive hormones in male smokers and the relationship of reproductive hormones with semen quality parameters in infertile smokers is inadequately understood.

This study aims to address these research gaps by evaluating the adverse effects of cigarette smoking on male reproductive health by analyzing the changes in semen quality and reproductive hormone levels. Further, the association of reproductive hormones with semen parameters has been evaluated using correlation and multiple linear regression regression analysis.

## 2. Materials and Methods

### 2.1. Study Population and Data Collection

This observational retrospective study, conducted between January 2023 and March 2025, involved 80 men experiencing infertility. Subjects were recruited from among couples visiting hospitals with difficulties in conception. Participants were divided into two groups based on smoking habits: 40 identified as smokers and 40 as non-smokers. Inclusion criteria for the study comprised the following factors: (1) age between 18 and 50 years; (2) sexual abstinence of 2–7 days; (3) absence of testicular injury, reproductive surgery, or prolonged use of any medications; and (4) absence of any diagnosed reproductive disorders. All participants signed informed consent, allowing the use of their information for scientific purposes. The study was conducted in accordance with the Declaration of Helsinki, and the ethical approval was obtained from the Institutional Ethics Committee of Assam University, Silchar (vide approval no. IEC/AUS/2021/35/BPC dated 26 February 2021). All participants filled up structured questionnaires, providing details on their demographic and lifestyle variables, including smoking history.

### 2.2. Semen Analysis and Hormone Assessment

Semen samples were obtained by masturbation and analyzed according to the latest World Health Organization (WHO) guidelines [25]. Serum levels of FSH, PRL, and total testosterone were analyzed using chemiluminescent immunoassay techniques (Beckman Coulter [Access 2] immunoassay system, Brea, CA, USA). LH was analyzed using the Standard F200-SD Biosensor (Suwon-Si, Republic of Korea) and the Standard F-LH fluorescent immunoassay (Gurugram, India).

### 2.3. Statistical Analysis

Statistical analyses were performed using Python (v 3.11.5; Python Software Foundation, 2023). Descriptive analyses were stratified according to smoking status. Normality was tested using the Shapiro–Wilk normality test. Normally distributed data (i.e., age) were compared using an independent t-test. The remaining parameters, which did not follow a normal distribution, were compared using the Mann–Whitney U test. Correlation and multiple linear regression analysis were performed for the complete dataset.

## 3. Results

### 3.1. Descriptive Statistics Comparing Age, Comorbidities, and Lifestyle Habits Between Smokers and Non-Smokers

In this study, the age of smokers (35.77 ± 5.89 years) and non-smokers (35.10 ± 6.53 years) was found to be almost similar, showing that the groups (smokers vs. non-smokers) were well matched in terms of age. The similar age of the two groups indicates that age is unlikely to confound the observed associations.

The incidence of COVID-19 was slightly more common among smokers (0.35 ± 0.48) than non-smokers (0.20 ± 0.41). Scientific evidence supports our findings, with reports showing that smoking increases the risk of COVID-19 by raising angiotensin-converting enzyme 2 (ACE2) receptors in airway cells, which damage lung tissues and increase susceptibility to infection [26]. Similarly, a meta-analysis conducted by Gülsen et al. also confirms the link between active smoking and increased vulnerability to COVID-19 infection [27]. Another recent meta-analysis revealed that semen parameters such as sperm count and motility are susceptible to damage by COVID-19 infection, although the influence of other confounding factors was not evaluated for such damage [28]. A higher prevalence of COVID-19 history may indicate increased susceptibility; however, the effect of COVID-19 on the semen cannot be confirmed from the present study.

Additionally, smokers exhibited slightly lower prevalance of diabetes (0.07 ± 0.27) than non-smokers (0.15 ± 0.36). Our finding contrasts with previous research, which have indicated a strong link between smoking and diabetes mellitus [29,30,31]. Given the limited sample size in our study, further research is required to explore the association.

Moreover, alcohol intake was reportedly higher in smokers (0.40 ± 0.50) than in non-smokers (0.28 ± 0.45), indicating a potential clustering of risk behaviors. Previous research indicates a strong negative link between alcohol intake and male fertility; alcohol has been shown to impair semen parameters [32]. Alcohol intake can affect male fertility through several mechanisms, including excessive reactive oxygen species (ROS) generation, OS, sperm DNA fragmentation, apoptosis, genotoxicity, inflammation leading to impairment of sperm cell production, disruption of hormonal balance, and decreased spermatogenesis [33]. Likewise, Amor et al. found that heavy alcohol consumption significantly reduces sperm quality and damages sperm DNA integrity, contributing to male infertility [34]. However, a study from the Netherlands with 163 participants confirms that alcohol consumption does not have any significant negative impact on semen parameters, such as volume, sperm count, motility, and morphology [35]. This discrepancy may stem from differences in study populations and alcohol consumption levels, highlighting the need for further research to better understand these effects.

In addition, smokers consumed slightly less tea and coffee (0.78 ± 0.42) than non-smokers (0.85 ± 0.36). Previous research suggests that tea consumption significantly improves sperm parameters, including concentration, total sperm count, and progressive motility [36]. In contrast, excessive coffee intake may harm male fertility by causing sperm DNA damage [37].

Furthermore, in this study, exposure to industry was more common among smokers (0.55 ± 0.50) as compared to non-smokers (0.25 ± 0.44), which could lead to confounding effects due to environmental co-exposures.. Our study emphasizes the need for further research to segregate the effects of smoking from industrial co-exposures, considering exposure duration and intensity to assess the potential impact of industrial exposure on male fertility.

Additionally, the average sleep duration was slightly longer in smokers (7.50 ± 1.06 h) than in non-smokers (7.25 ± 0.90 h) (Supplementary Table S1). Although smokers reported slightly longer sleep durations, the clinical significance of the difference remains unclear. On the contrary, a meta-analysis reported shortened sleeping duration in smokers, possibly due to the interference of nicotine-induced release of dopamine [38]. Based on our results, it is plausible that increased PRL levels (discussed in the next section) counteract dopamine-induced excitatory effects in smokers. Nonetheless, further validation in larger populations is essential to substantiate this finding.

### 3.2. Descriptive Statistics Comparing Semen Parameters and Serum Hormone Levels Between Smokers and Non-Smokers

Smokers had a slightly longer abstinence period (3.12 ± 0.33 days) compared to non-smokers (3.00 ± 0.32 days), although this difference is unlikely to have significant clinical implications.

Smokers showed a slightly lower semen volume (1.85 ± 0.92 mL) than non-smokers (1.97 ± 0.87 mL). Our findings align with previous reports, indicating reduced semen volume in smokers [39,40]. Henriques et al. observed similar outcomes in their investigation, indicating that smoking was associated with diminished semen volume and a lower total sperm count [17]. However, Saaranen et al. have demonstrated that although heavy smokers have significantly lower semen volume, occasional and regular smokers have semen volumes comparable to those observed in non-smokers [41]. Similarly, current smokers showed lower semen volume compared to past smokers and never smokers [42]. It has also been shown that quitting smoking significantly improves semen volume among infertile heavy smokers, suggesting that the smoking effect on accessory gland function is reversible [43]. It has been suggested that the decline in semen volume in smokers may be attributed to the impact of nicotine on the accessory glands, such as seminal vesicles, prostate, and urethral glands [17,43].

Liquefaction time was slightly faster in smokers (24.98 ± 10.38 min) compared to non-smokers (25.95 ± 9.92 min), although the difference was minimal. Research shows that semen liquefaction time generally does not differ significantly between smokers and non-smokers [44,45]. However, another study reported that smokers who consumed more than 10 cigarettes daily experienced longer liquefaction times [46].

The seminal pH was slightly lower in smokers (7.32 ± 0.44) than in non-smokers (7.42 ± 0.36), possibly indicating changes in seminal plasma buffering, although the difference is not statistically significant. Previous studies also suggest that smoking does not affect seminal pH in infertile subjects [39,47,48].

Pus cell concentration was higher in smokers (0.46 ± 0.72 × 10^6^/mL) than in non-smokers (0.29 ± 0.54 × 10^6^/mL). Our results align with earlier research reporting increased leukocyte counts in the semen of infertile smokers [39,49]. This points to an increased risk of inflammation or infection [50], though the mechanisms behind leukocyte activation in smoker semen remain unclear.

Live sperm percentages were significantly lower in smokers (32.23 ± 15.94%) compared to non-smokers (60.83 ± 19.26%), while the percentage of dead sperm cells was higher (65.28% vs. 31.68%), reflecting the negative impact of smoking on sperm viability. Studies have demonstrated that smoking worsens sperm vitality among infertile men [51] and heavy tobacco smokers [52]. Additionally, sperm vitality has been shown to be compromised in fertile smokers [50]. Conversely, Brucker et al. (2020) found no significant difference in sperm vitality between infertile smokers and non-smokers [53]. Motile sperm cells (%) were also reduced in smokers (46.12 ± 28.86%) compared to non-smokers (50.95 ± 26.26%), suggesting impaired motility. Our findings concur with other studies showing reduced motility and an increased percentage of non-motile sperm in smokers [39,52]. Notably, Ranganathan et al. observed that sperm motility declines significantly among infertile smokers compared to fertile smokers [47]. Another study reported that heavy smokers exhibit a lower average percentage of motile spermatozoa compared to non-smokers [6].

Both sperm concentration and total sperm count were lower in smokers (28.75 ± 28.54 × 10^6^/mL and 63.00 ± 80.80 × 10^6^ per ejaculation) compared to non-smokers (38.16 ± 33.07 × 10^6^/mL and 78.17 ± 101.72 × 10^6^ per ejaculation). Consistent with our findings, previous studies have reported the overall harmful effects of smoking on sperm concentration and total sperm count. Bundhun et al. had demonstrated that tobacco smoking is associated with lower sperm count and increased morphological defects [48]. Similarly, Brucker et al., in a large retrospective study, found a significant decline in sperm concentration in infertile smokers [53]. Lewin et al. also reported reduced sperm concentration in infertile smokers, although no statistically significant differences were observed for sperm motility [54]. A recent study has indicated that moderate or heavy smoking affects sperm concentration and motility in infertile men [55]. However, Trummer et al. reported no significant differences in sperm concentration or motility between smokers, ex-smokers, and non-smokers [49]. Interestingly, another study highlighted improvements in sperm concentration and total sperm count following three months of smoking cessation [43]. The decline in total sperm count may be attributed to decreased semen volume in smokers [17].

Normal sperm morphology was lower in smokers (2.10 ± 1.55%) than in non-smokers (2.90 ± 1.77%), while sperm head abnormalities were more common among smokers (27.70 ± 20.14%) compared to non-smokers (34.27 ± 17.83%). No significant differences were seen in neck and tail abnormalities. Additionally, the proportion of multiple sperm morphological abnormalities was slightly lower in smokers (22.62 ± 17.94%) than in non-smokers (24.75 ± 17.40%). Our results are consistent with a previous cross-sectional study, which found no statistically significant difference in normal morphology between smokers and non-smokers, yet reported a decline in normal morphology among smokers [17]. Another study reported no significant difference in sperm concentration among light/moderate smokers, heavy smokers, and non-smokers [56]. Similarly, Trummer et al. found no significant differences in sperm morphology among smokers, ex-smokers, and non-smokers [49]. However, a recent meta-analysis involving 10,823 infertile individuals stratified by smoking status revealed a higher prevalence of sperm morphological abnormalities among smokers, with notable increases in head, neck, and tail defects [48]. Another study found a significant correlation between heavy/moderate smoking and abnormal sperm morphology [55].

Smokers showed significantly higher levels of FSH (10.37 ± 4.58) and LH (8.95 ± 1.42) compared to non-smokers (5.93 ± 2.67 and 5.64 ± 2.67 respectively) (*p* = 0.0000), which may indicate impaired testicular function and compensatory hormonal feedback. Moreover, PRL levels were notably elevated in smokers (14.05 ± 3.87 ng/mL) as compared to non-smokers (7.04 ± 2.24 ng/mL) (*p* = 0.0000). Testosterone levels were slightly lower in smokers (4.19 ± 2.12 ng/mL) than in non-smokers (4.53 ± 2.43 ng/mL), though this difference was not statistically significant (Table S1). Our study aligns with the findings of Mendelson et al., where high nicotine cigarette smoke was associated with a rise in LH and PRL levels without any significant change in the levels of testosterone [22]. Our findings are further consistent with earlier studies which reported elevated LH levels among smokers; however, the observed increase in testosterone contradicts our results [6]. Similar findings were reported by Trummer et al., who observed increased LH and testosterone in smokers but also noted decreased PRL levels among smokers, which contradicts our data [49]. Another study found no statistical differences in FSH, LH, PRL, or testosterone levels [48]. Yet another research revealed an inverse correlation between FSH, LH, testosterone, and sperm motility, as well as an inverse association of FSH and LH with sperm concentration [57]. Our findings also support a study showing higher FSH and LH, with lower testosterone levels in infertile heavy smokers [24]. PRL concentrations have been shown to rise proportionally with the number of cigarettes smoked per day in active smokers [58]. Higher levels of PRL are attributed to the smoking-induced inhibition of dopamine regulation [59], which might be another reason for the dysregulation of the hypothalamic–pituitary–gonadal (HPG) axis in smokers. As the earlier studies have demonstrated that in vitro administration of dopamine can enhance sperm motility and vitality due to its antioxidant effects [60,61]. Therefore, dopamine inhibition may impact sperm motility and vitality; however, further investigation is necessary to elucidate the underlying mechanisms.

### 3.3. Semen Parameters and Their Interrelationships

#### 3.3.1. Positive Correlation Between Sperm Variables

**Figure 1:**
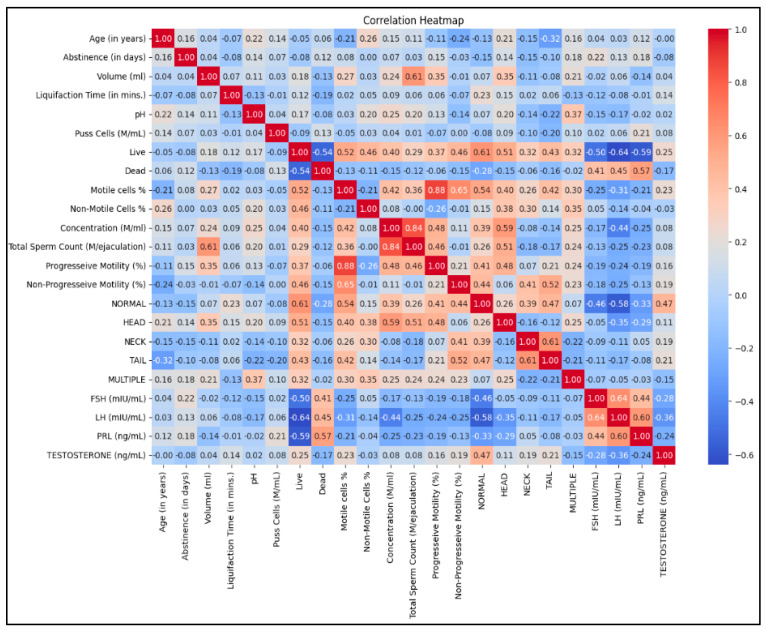
A correlation heatmap illustrating the relationships between semen analysis results and hormone variables among the participants.

The correlation heatmap highlights several strong positive relationships among semen quality variables. Notably, the percentage of motile sperm cells correlates highly with sperm cell progressive motility (r = 0.88), suggesting that sperm motility is largely driven by sperm cell forward movement. Similarly, the live sperm cell percentage shows a strong correlation with the motile sperm cell percentage (r = 0.65), indicating that viable sperm cells are more likely to be motile. The total sperm count also correlates strongly with sperm concentration (r = 0.84), as expected, because the total sperm count depends on both concentration and ejaculate volume. These findings confirm the consistency among sperm functional parameters (Figure 1).

#### 3.3.2. Negative Correlations with Sperm Viability

There are notable negative correlations between dead sperm cells and other sperm parameters. For instance, the percentage of live cells is strongly inversely related to the percentage of dead cells (r = −0.54), highlighting their opposing trends. Likewise, the percentages of motile cells and progressive motility exhibit moderate-to-strong negative correlations with dead cells (r = −0.52 and −0.51, respectively). This suggests that lower semen viability is often associated with decreased sperm motility and function, which may impact fertility (Figure 1).

#### 3.3.3. Hormonal Influences on Semen Quality

The heatmap (Figure 1) also highlights interactions between reproductive hormones and semen quality parameters. Notably, FSH is negatively correlated with live cells (r = −0.50) and total sperm count (r = −0.46), indicating that elevated FSH levels may reflect impaired spermatogenesis, often associated with testicular dysfunction. A similar trend is seen with PRL, which has a strong negative correlation with live cells (r = −0.59) and sperm concentration (r = −0.50). On the other hand, testosterone exhibits a modest positive correlation with normal sperm morphology (r = 0.47), suggesting a supportive role in maintaining morphological integrity.

#### 3.3.4. Interrelationship Among Sperm Morphological Forms

Within sperm morphology, specific structural correlations are clear. The percentage of normal morphology is positively correlated with head morphology (r = 0.62), indicating that abnormalities in the head region have a significant impact on overall morphology classification. Additionally, multiple defects are negatively associated with normal morphology (r = −0.58), showing that morphological anomalies tend to cluster across different forms. These insights could aid in targeted morphological assessment during diagnostic evaluations.

#### 3.3.5. Abstinence, Age, Liquefaction, and pH

The duration of abstinence has a moderate correlation with ejaculate volume (r = 0.35), suggesting that longer abstinence could increase semen volume. Meanwhile, age shows only a weak correlation with most parameters, indicating that age-related effects are likely subtle or nonlinear within this sample. Liquefaction time and pH exhibit minimal links to fertility-related factors.

### 3.4. Comparative Analysis of Semen Quality Parameters and Serum Reproductive Hormone Levels in Smokers Versus Non-Smokers

The Shapiro–Wilk normality test showed that only age (*p* = 0.0684) follows a normal distribution among all measured parameters. All other variables significantly deviate from normality (*p* < 0.05), including abstinence duration, semen volume, liquefaction time, pH, pus cells, live/dead sperm cells count (vitality), and motile, non-motile, progressive, and non-progressive, as well as morphological traits like normal forms, head defects, neck defects, tail, and multiple abnormalities. Additionally, hormonal markers such as FSH, LH, PRL, and testosterone also display non-normal distributions (Table S2). These findings suggest considerable biological variability and potential skewness in the reproductive profiles of the study group. Consequently, the Mann–Whitney U test, a non-parametric statistical method, is more suitable for comparative and inferential analyses of this dataset. The comparison revealed no significant difference in age distribution between smokers and non-smokers (*p* = 0.6289), indicating that they are well matched in terms of age. Liquefaction time (*p* = 0.5795), semen volume (*p* = 0.2940), and pH (*p* = 0.0683) also showed no significant differences between the groups. Our findings align with Singh and Thotakura (2018), who also found no difference in liquefaction time between smokers and non-smokers [62]. Similarly, Meri et al. reported that semen volume and pH levels remain unchanged in smokers, indicating that smoking does not affect these basic semen parameters [63]. Conversely, Nematollahi–Mahani et al. observed significant differences, including shorter liquefaction time, decreased semen volume, and higher pH levels in smokers compared to non-smokers [64]. The discrepancies in our findings may be due to our small sample size, which limits the statistical power to detect differences between smokers and non-smokers. Therefore, further research involving a larger sample size is necessary to understand the underlying mechanism of smoking’s effect on the above-mentioned semen parameters.

Significant differences were observed in sperm vitality between smokers and non-smokers. The proportion of live sperm was significantly lower (*p* = 0.0000), while the proportion of dead sperm was significantly higher (*p* = 0.0000) in smokers, indicating that smoking adversely affects sperm viability. Toxins from cigarette smoke increase ROS levels, leading to OS that damages sperm cell membranes. They target the polyunsaturated fatty acids within these membranes, compromising their integrity and decreasing sperm viability [16]. However, no significant differences were found in total motility (*p* = 0.4577), non-motile sperm percentage (*p* = 0.4291), progressive motility (*p* = 0.7613), or non-progressive motility (*p* = 0.3821), indicating that while viability is impacted, the motility patterns of surviving sperm are similar between groups. Our findings are supported by similar studies where no significant difference was observed in sperm motility between infertile smokers and non-smokers [48,65].

No significant differences were found in sperm concentration (*p* = 0.2457) or total sperm count per ejaculation (*p* = 0.2638), suggesting that smoking may not significantly alter total sperm production, although it does affect sperm vitality. Similarly, the number of pus cells showed no significant variation between groups (*p* = 0.2706), indicating infection-related parameters are not strongly influenced by smoking within this sample population. Moreover, morphological assessment revealed a significant difference in the percentage of normal sperm forms (*p* = 0.0302), with smokers exhibiting lower values. Other morphological defects, such as head (*p* = 0.0836), neck (*p* = 0.7869), tail (*p* = 0.5653), and multiple abnormalities (*p* = 0.6197), were not significantly different. This suggests smoking mainly affects the overall proportion of morphologically normal sperm rather than specific defect types [66].

The hormonal profile comparison revealed significant differences for several reproductive hormones. Levels of FSH (*p* = 0.0000) and LH (*p* = 0.0000) were significantly higher in smokers, possibly reflecting a compensatory response of the HPG axis to impaired spermatogenesis. PRL also differed significantly (*p* = 0.0000), whereas testosterone levels showed no significant difference (*p* = 0.5539) (Table S3). Our findings follow the works of Wilkins et al., who reported higher PRL levels in chronic smokers [59]. In contrast, Trummer et al. reported lower levels of PRL in smokers as compared to non-smokers [49]. Interestingly, Ochedalski et al. observed lower levels of LH, FSH, and PRL, and unaltered levels of testosterone in smokers when compared with non-smokers [67]. The study by Mitra et al., however, showed higher FSH, LH, and lower testosterone levels in smokers [24]. The mechanistic view for these interactions may be derived from the study of Blanco-Muñoz et al., where it has been shown that smokers had increased LH, PRL, and testosterone levels [68], which could be a result of compensatory upregulation of the HPG axis in response to nicotine-induced gonadal impairment or altered dopaminergic/serotonergic activities [59].

The concurrent elevations of LH, FSH, and PRL in smokers, despite unchanged testosterone, may be a result of central hypothalamic–pituitary stimulation with possible Leydig cell compensation. This needs further analysis with larger cohorts.

Therefore, the findings indicate that smoking is significantly associated with reduced sperm vitality and normal morphology, along with changes in key reproductive hormone levels (FSH, LH, and PRL). However, other parameters, including motility, sperm count, testosterone levels, and most morphological defects, were not significantly impacted.

### 3.5. Key Variables Influenced by Smoking

In this study, the variables such as live sperm cells (%), dead sperm cells (%), normal sperm cell morphology (%), FSH (mIU/mL), LH (mIU/mL), and PRL (ng/mL) are the key parameters for assessing male fertility and reproductive health. These measures serve as indicators of semen quality and reflect the endocrine function, providing valuable insights into how lifestyle factors like smoking may affect male reproductive health and fertility.

**Figure 2:**
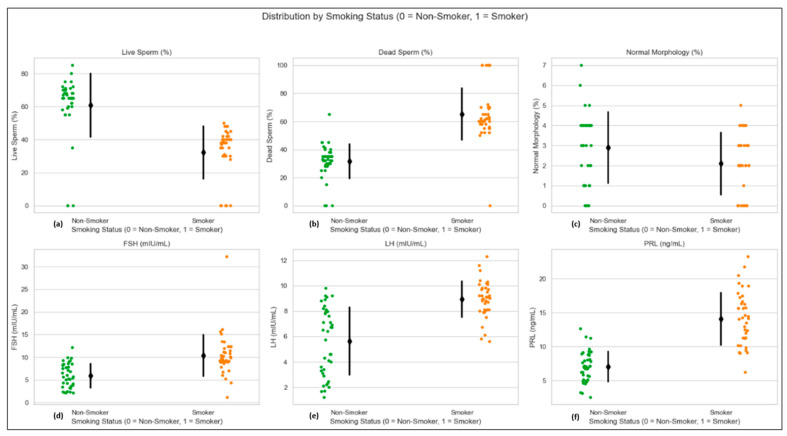
This figure illustrates the differences in (**a**) live sperm cells (%), (**b**) dead sperm cells (%), (**c**) normal morphology (%), (**d**) FSH (mIU/mL), (**e**) LH (mIU/mL), and (**f**) PRL (ng/mL) between smoker and non-smoker groups. Each subplot demonstrates a significant decline in semen quality and abnormal hormonal levels among smokers, emphasizing the adverse impact of tobacco use on male reproductive health. This analysis underscores the negative effects of smoking on reproductive parameters, providing a comprehensive scientific evaluation.

The scatter plot depicting the distribution of live sperm percentage between smokers and non-smokers shows a stark contrast (Figure 2a). Non-smokers consistently exhibit higher live sperm percentages, mostly clustered above 60%, with several individuals reaching up to 80%. In contrast, smokers typically show much lower values, usually ranging from 30% to 45%, with some cases even lower than 0%. This visual disparity highlights a clear negative impact of smoking on sperm viability. The presence of more outliers near zero in the smoking group suggests increased sperm cell mortality, likely due to OS and toxic compounds in tobacco smoke, which impairs spermatogenesis.

In contrast, Figure 2b illustrates the inverse relationship with live sperm percentage. Smokers show a consistently higher proportion of dead sperm compared to non-smokers. While non-smokers generally have dead sperm cell percentages below 30%, smokers often exceed this threshold. The increase in dead sperm cells among smokers indicates enhanced sperm degradation and cellular apoptosis, potentially caused by chronic exposure to reactive oxygen species (ROS) and genotoxic substances in cigarette smoke. This supports the hypothesis that smoking directly impairs semen quality.

Although differences in sperm morphology between smokers and non-smokers are evident, they appear less pronounced than those observed in viability metrics. Non-smokers tend to have a slightly higher percentage of sperm with normal morphology, with values typically concentrated between 40% and 55%. Smokers, on the other hand, often show lower morphology percentages, indicating a larger proportion of abnormal sperm cells (Figure 2c). These morphological abnormalities may impair motility and the sperm’s ability to fertilize an egg, suggesting that even moderate shifts in this parameter are clinically relevant. On the other hand, the plot for FSH shows elevated levels in smokers (Figure 2d). Non-smokers typically have FSH values within a normal range, while smokers display greater variation and higher average levels. Since FSH is a pituitary hormone that regulates sperm production, its increase in smokers could be a compensatory response to declining testicular function. Similarly, LH levels also show a slight increase among smokers (Figure 2e). This hormone is crucial for testosterone regulation through its action on Leydig cells. The graph indicates that smokers tend to have more dispersed and elevated LH values, suggesting possible disruption in the HPG axis. This hormonal imbalance may result from decreased Leydig cell function due to long-term exposure to nicotine and other toxins from smoking. The plot for PRL reveals higher concentrations in smokers compared to non-smokers (Figure 2f). Although PRL is not a primary gonadotropin, its increase can interfere with the normal pulsatility of GnRH and suppress both LH and FSH, affecting fertility. The trend in the graph indicates that non-smokers maintain relatively stable and lower PRL levels, whereas smokers exhibit greater variation and higher peaks. Elevated PRL in smokers could be due to stress-related endocrine disruption or impaired dopaminergic control, both of which can impact reproductive output [69].

### 3.6. Relationship Between Hormonal Profiles and Semen Parameters: A Multiple Linear Regression Approach

We performed regression analyses to explore the relationships between various hormonal predictors and measures of sperm quality, including sperm concentration, total sperm count, sperm morphology, total motility, progressive motility, and sperm vitality (percentage of live and dead sperm) among the participants in our study. The results showed that higher levels of LH are linked to lower sperm concentration (R^2^ = 22.3%; *p* < 0.001). No other hormones showed a statistically significant relationship with sperm concentration in this dataset.

**Figure 3:**
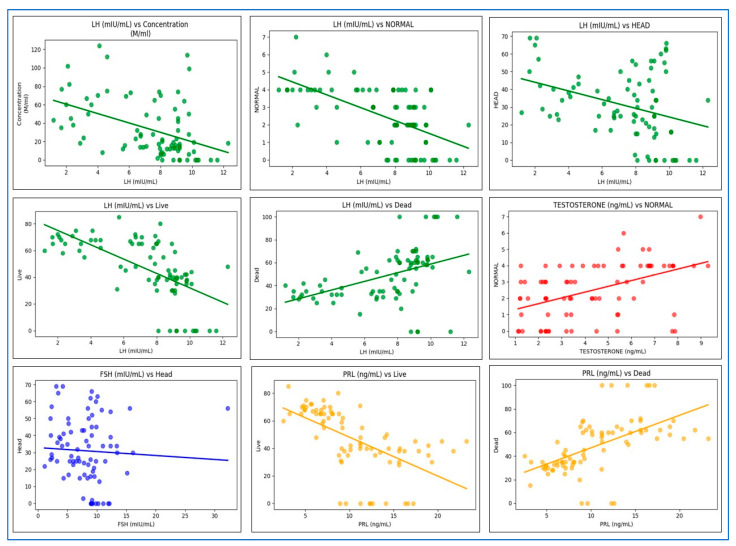
The regression coefficients and 95% confidence intervals of hormonal predictors (FSH, LH, PRL, and testosterone) in relation to key sperm quality variables reveal nuanced impacts of reproductive hormones on sperm characteristics. Notably, LH is consistently recognized as a negative predictor of sperm concentration, morphology, and vitality, highlighting its detrimental effects when elevated. In contrast, testosterone positively impacts normal sperm morphology, emphasizing its essential role in maintaining structural integrity. While FSH demonstrates a less pronounced overall effect, it is significantly linked to an increase in head defects. Prolactin exhibits a strong association with heightened sperm motility and reduced vitality, indicating its suppressive influence on sperm function. These findings underscore the hormone-specific regulatory functions in male fertility, positioning LH and PRL as critical markers of compromised semen quality.

Our findings are consistent with the results of a previous study conducted by Meeker et al., which reported the inverse relationship between LH and sperm concentration. Elevated LH levels may serve as a compensatory mechanism in response to decreased Leydig cell activity and lower testosterone production [69]. When Leydig cells are hypoactive, the anterior pituitary increases LH secretion to support spermatogenesis and testosterone synthesis. However, if Leydig cells are severely damaged or unresponsive to LH, as shown in Figure 3, this increase in LH might not be sufficient to restore adequate testosterone levels needed for normal spermatogenesis, which can hinder the process and thereby reduce sperm concentration [70,71].

Regarding the total sperm count, no hormone emerged as a significant predictor, leaving most of the variability unexplained (R^2^ = 7.5%). Furthermore, higher LH levels were associated with poorer sperm morphology, while increased testosterone levels were correlated with better sperm morphology (*p* < 0.01). Interestingly, elevated LH levels are also linked to fewer head defects (*p* < 0.01). This suggests that other morphological deformities, including those of the neck, tail, and multiple defects, are more common in this population.

Previous research also found a connection between elevated LH levels and poor sperm morphology [57]. This could arise from LH’s impact on sperm metabolic functions and the degeneration of seminiferous tubules, which results in reduced testosterone production. Lower testosterone impairs normal sperm development, leading to various sperm deformities [72,73]. Conversely, higher serum FSH levels were linked to increased head defects (*p* < 0.05). An inverse relationship between FSH and sperm morphology has been observed, possibly due to sperm DNA fragmentation and abnormal spermatogenesis [60].

Furthermore, both higher LH and higher PRL levels were associated with lower percentages of live sperm cells (*p* < 0.01) and an increase in the percentage of dead sperm cells. This may be due to OS-mediated sperm DNA damage, impairment of sperm cell membrane integrity [74], which ultimately contributes to sperm cell apoptosis. The elevation of prolactin levels has a significant impact on male reproductive function as it suppresses the pulsatile secretion of GnRH from the hypothalamus. This suppression leads to decreased release of LH and FSH from the pituitary gland. Both LH and FSH are essential for stimulating testosterone production and maintaining the normal spermatogenesis process. When the levels of these hormones decline, this can significantly impair spermatogenesis, ultimately reducing overall sperm vitality and fertility in men [66]; however, the exact mechanism is not clear.

Notably, elevated testosterone levels exerted a positive influence, demonstrating a significant relationship with a higher proportion of morphologically normal sperm, suggesting a supportive role in spermatogenesis and overall sperm health.

**Figure 4:**
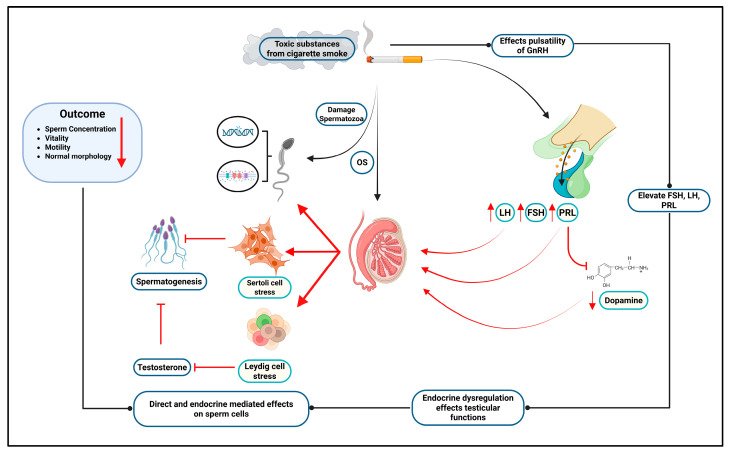
The effect of cigarette smoking on sperm quality mediated via hormonal imbalance. Toxins from cigarette smoke affect GnRH secretion, which dysregulates the release of luteinizing hormone (LH) and follicle-stimulating hormone (FSH). Additionally, dopaminergic control of prolactin (PRL) is impaired, thereby elevating PRL secretion. Elevated LH and FSH induce testicular damage, disrupt testosterone homeostasis, and inhibit spermatogenesis. OS = oxidative stress.

FSH primarily affected sperm head abnormalities but did not significantly impact other parameters. Notably, total sperm count, neck and tail morphological abnormalities, and non-progressive motility were not substantially associated with hormonal profiles alone, suggesting the involvement of additional determinants in male reproductive function. Overall, our findings underscore the importance of hormonal balance—particularly concerning LH, PRL, and testosterone—in maintaining optimal sperm quality while also highlighting the need to consider other influences for a comprehensive understanding of male infertility (Figure 4).

However, the present study is not free from limitations, including self-reported data on smoking habits and other sociodemographic and lifestyle parameters, which might have introduced reporting bias, potentially influencing the study outcomes. Furthermore, the study outcomes may have been confounded by simultaneous exposure to multiple environmental agents, including EDCs, whose potential impact was not incorporated into the present analysis. Additionally, specific metrics such as the concentration of smoking-derived metabolites and the number of cigarettes smoked per unit time were not incorporated into the present analysis.

## 4. Conclusions

This study holds dual significance, which highlights the detrimental effects of smoking on both sperm quality and reproductive hormone profiles, and offers a comprehensive view of tobacco-induced male reproductive dysfunction. Our findings highlight the possible hormonal crosstalk that exaggerates the impact of smoking on semen quality. In this study, smokers showed significantly lower percentages of live spermatozoa along with notable differences in reproductive hormone profiles. Hormonal profiling revealed elevated levels of FSH, LH, and PRL in smokers as compared to non-smokers, offering important insight into testicular dysfunction caused by smoking. Higher FSH and LH likely disrupt the negative feedback loop, thereby impairing the normal functioning of the HPG axis and spermatogenesis. Additionally, elevated PRL could interfere with gonadotropin signaling (FSH and LH), raising questions about the role of dopamine regulation. Further studies with larger sample sizes should include confounding hormones to address gaps and deepen understanding of the hormonal mechanisms involved in infertility.

## Data Availability

The original contributions presented in this study are included in the article.

## Supplementary Materials

The following supporting information can be downloaded at: https://www.mdpi.com/article/10.3390/cells14171345/s1, Table S1: Descriptive statistics analysis; Table S2: Normality test using Shapiro–Wilk test; Table S3: Statistical comparison between smokers and non-smokers.

## Abbreviations

The following abbreviations are used in this manuscript:

WHO	World Health Organization
FSH	Follicle-stimulating hormone
LH	Luteinizing hormone
PRL	Prolactin
DNA	Deoxyribonucleic acid
OS	Oxidative stress
ROS	Reactive oxygen species
HPG	Hypothalamic–pituitary–gonadal (axis)
EDC	Endocrine-disrupting chemical
PCB	Polychlorinated biphenyls
PBB	Polybrominated biphenyl
BPA	Bisphenol A
DDT	Dichlorodiphenyltrichloroethane
