# Supplementary material for: Endocrine Disruption as a Mediator of Declining Semen Quality in Smokers
Source: Cells. 2025 Aug 29;14(17):1345. doi: 10.3390/cells14171345 (PMC12428148; doi:10.3390/cells14171345)
Supplement: Supplementary file 1 [file cells-14-01345-s001.zip › cells-3809908-supplementary.pdf]

cells

MDPI

# Supplementary File S1

**Table S1: Descriptive Statistics Analysis**

| **Parameters** | **Non-Smoker** (Mean ± **SD)** | **Smoker** (Mean ± **SD)** |
|---|---|---|
| **Age (in years)** | 35.10 ± 6.53 | 35.77 ± 5.89 |
| **Covid-19** | 0.20 ± 0.41 | 0.35 ± 0.48 |
| **Diabetes** | 0.15 ± 0.36 | 0.07 ± 0.27 |
| **Tobacco** | 0.42 ± 0.50 | 0.42 ± 0.50 |
| **Alcohol** | 0.28 ± 0.45 | 0.40 ± 0.50 |
| **Tea/Coffee** | 0.85 ± 0.36 | 0.78 ± 0.42 |
| **Industry** | 0.25 ± 0.44 | 0.55 ± 0.50 |
| **Sleep** | 7.25 ± 0.90 | 7.50 ± 1.06 |
| **Abstinence (in days)** | 3.00 ± 0.32 | 3.12 ± 0.33 |
| **Volume (ml)** | 1.97 ± 0.87 | 1.85 ± 0.92 |
| **Liquefaction Time (in mins.)** | 25.95 ± 9.92 | 24.98 ± 10.38 |
| **pH** | 7.42 ± 0.36 | 7.32 ± 0.44 |
| **Puss Cells (M/mL)** | 0.29 ± 0.54 | 0.46 ± 0.72 |
| **Live** | 60.83 ± 19.26 | 32.23 ± 15.94 |
| **Dead** | 31.68 ± 12.12 | 65.28 ± 18.41 |
| **Motile cells %** | 50.95 ± 26.26 | 46.12 ± 28.86 |
| **Non-Motile Cells %** | 41.55 ± 24.84 | 36.38 ± 25.65 |
| **Concentration (M/ml)** | 38.16 ± 33.07 | 28.75 ± 28.54 |
| **Total Sperm Count (M/ejaculation)** | 78.17 ± 101.72 | 63.00 ± 80.80 |
| **Progressive Motility (%)** | 30.57 ± 21.48 | 29.12 ± 21.89 |

 https://doi.org/10.3390/cells14171345

| | | |
|---|---|---|
| **Non-Progressive Motility (%)** | 20.38 ± 14.50 | 17.18 ± 12.38 |
| **NORMAL** | 2.90 ± 1.77 | 2.10 ± 1.55 |
| **HEAD** | 34.27 ± 17.83 | 27.70 ± 20.14 |
| **NECK** | 16.18 ± 11.66 | 17.05 ± 16.32 |
| **TAIL** | 14.40 ± 11.11 | 13.03 ± 11.95 |
| **MULTIPLE** | 24.75 ± 17.40 | 22.62 ± 17.94 |
| **FSH (mIU/mL)** | 5.93 ± 2.67 | 10.37 ± 4.58 |
| **LH (mIU/mL)** | 5.64 ± 2.67 | 8.95 ± 1.42 |
| **PRL (ng/mL)** | 7.04 ± 2.24 | 14.05 ± 3.87 |
| **TESTOSTERONE (ng/mL)** | 4.53 ± 2.43 | 4.19 ± 2.12 |

**Table S2: Normality Test using Shapiro–Wilk Test**

| **Parameter** | **p-value** | **Normality** |
|---|---|---|
| **Age (in years)** | 0.0684 | Normal |
| **Abstinence (in days)** | 0.0000 | Not Normal |
| **Volume (ml)** | 0.0000 | Not Normal |
| **Liquefaction Time (in mins.)** | 0.0002 | Not Normal |
| **pH** | 0.0000 | Not Normal |
| **Pus Cells (M/mL)** | 0.0000 | Not Normal |
| **Live** | 0.0000 | Not Normal |
| **Dead** | 0.0020 | Not Normal |
| **Motile cells %** | 0.0002 | Not Normal |
| **Non-Motile Cells %** | 0.0041 | Not Normal |
| **Concentration (M/ml)** | 0.0000 | Not Normal |
| **Total Sperm Count (M/ejaculation)** | 0.0000 | Not Normal |
| **Progressive Motility (%)** | 0.0003 | Not Normal |
| **Non-Progressive Motility (%)** | 0.0011 | Not Normal |

| | | |
|---|---|---|
| **NORMAL** | 0.0000 | Not Normal |
| **HEAD** | 0.0180 | Not Normal |
| **NECK** | 0.0000 | Not Normal |
| **TAIL** | 0.0000 | Not Normal |
| **MULTIPLE** | 0.0009 | Not Normal |
| **FSH (mIU/mL)** | 0.0000 | Not Normal |
| **LH (mIU/mL)** | 0.0000 | Not Normal |
| **PRL (ng/mL)** | 0.0136 | Not Normal |
| **TESTOSTERONE (ng/mL)** | 0.0010 | Not Normal |

**Table S3: Statistical Comparison between Smokers and Non-Smokers**

| Parameter | Test Used | p-value | Significance (α = 0.05) |
|---|---|---|---|
| Age (in years) | T-test | 0.6289 | Not Significant |
| Abstinence (in days) | Mann–Whitney U | 0.0971 | Not Significant |
| Volume (ml) | Mann–Whitney U | 0.2940 | Not Significant |
| Liquefaction Time (in mins.) | Mann–Whitney U | 0.5795 | Not Significant |
| pH | Mann–Whitney U | 0.0683 | Not Significant |
| Pus Cells (M/mL) | Mann–Whitney U | 0.2706 | Not Significant |
| Live | Mann–Whitney U | **0.0000** | **Significant** |
| Dead | Mann–Whitney U | **0.0000** | **Significant** |
| Motile cells % | Mann–Whitney U | 0.4577 | Not Significant |
| Non-Motile Cells % | Mann–Whitney U | 0.4291 | Not Significant |
| Concentration (M/ml) | Mann–Whitney U | 0.2457 | Not Significant |
| Total Sperm Count (M/ejaculation) | Mann–Whitney U | 0.2638 | Not Significant |
| Progressive Motility (%) | Mann–Whitney U | 0.7613 | Not Significant |
| Non-Progressive Motility (%) | Mann–Whitney U | 0.3821 | Not Significant |

| | | | |
|---|---|---|---|
| NORMAL | Mann–Whitney U | **0.0302** | **Significant** |
| HEAD | Mann–Whitney U | 0.0836 | Not Significant |
| NECK | Mann–Whitney U | 0.7869 | Not Significant |
| TAIL | Mann–Whitney U | 0.5653 | Not Significant |
| MULTIPLE | Mann–Whitney U | 0.6197 | Not Significant |
| FSH (mIU/mL) | Mann–Whitney U | **0.0000** | **Significant** |
| LH (mIU/mL) | Mann–Whitney U | **0.0000** | **Significant** |
| PRL (ng/mL) | T-test | **0.0000** | **Significant** |
| TESTOSTERONE (ng/mL) | Mann–Whitney U | 0.5539 | Not Significant |

**Disclaimer/Publisher's Note:** The statements, opinions and data contained in all publications are solely those of the individual author(s) and contributor(s) and not of MDPI and/or the editor(s). MDPI and/or the editor(s) disclaim responsibility for any injury to people or property resulting from any ideas, methods, instructions or products referred to in the content.
